# Trehalose catalytic shift is an intrinsic factor in Mycobacterium tuberculosis that enhances phenotypic heterogeneity and multidrug resistance

**DOI:** 10.21203/rs.3.rs-4999164/v1

**Published:** 2024-09-13

**Authors:** Hyungjin Eoh, Jae Jin Lee, Daniel Swanson, Sun-Kyung Lee, Stephanie Dihardjo, Gi Yong Lee, Gelle Sree, Emily Maskill, Zachary Taylor, Michael Van Nieuwenhze, Abhyudai Singh, Jong-Seok Lee, Seok-Yong Eum, Sang-Nae Cho, Benjamin Swarts

**Affiliations:** University of Southern California; University of Southern California; Central Michigan University; Internal Tuberculosis Research Center; University of Southern California; University of Southern California; University of Southern California; Central Michigan University; Indiana University Bloomington; Baylor University; University of Delaware; International Tuberculosis Research Center; Internal Tuberculosis Research Center; Yonsei University College of Medicine; Central Michigan University

## Abstract

Drug-resistance (DR) in many bacterial pathogens often arises from the repetitive formation of drug-tolerant bacilli, known as persisters. However, it is unclear whether *Mycobacterium tuberculosis* (Mtb), the bacterium that causes tuberculosis (TB), undergoes a similar phenotypic transition. Recent metabolomics studies have identified that a change in trehalose metabolism is necessary for Mtb to develop persisters and plays a crucial role in metabolic networks of DR-TB strains. The present study used Mtb mutants lacking the trehalose catalytic shift and showed that the mutants exhibited a significantly lower frequency of the emergence of DR mutants compared to wildtype, due to reduced persister formation. The trehalose catalytic shift enables Mtb persisters to survive under bactericidal antibiotics by increasing metabolic heterogeneity and drug tolerance, ultimately leading to development of DR. Intriguingly, rifampicin (RIF)-resistant bacilli exhibit cross-resistance to a second antibiotic, due to a high trehalose catalytic shift activity. This phenomenon explains how the development of multidrug resistance (MDR) is facilitated by the acquisition of RIF resistance. In this context, the heightened risk of MDR-TB in the lineage 4 HN878 W-Beijing strain can be attributed to its greater trehalose catalytic shift. Genetic and pharmacological inactivation of the trehalose catalytic shift significantly reduced persister formation, subsequently decreasing the incidence of MDR-TB in HN878 W-Beijing strain. Collectively, the trehalose catalytic shift serves as an intrinsic factor of Mtb responsible for persister formation, cross-resistance to multiple antibiotics, and the emergence of MDR-TB. This study aids in the discovery of new TB therapeutics by targeting the trehalose catalytic shift of Mtb.

## Introduction

The World Health Organization (WHO) estimated that between 2000 and 2020, over 200 million people were ill with tuberculosis (TB), and more than 35 million died from the disease.^1^ In 2023, one fourth of world’s population was still infected with *Mycobacterium tuberculosis* (Mtb), the bacterium that causes TB. Conventional TB treatment typically involves the administration of multiple antibiotics over a prolonged period, sometimes extending up to two years. This extended duration is largely due to the wide range of antibiotic tolerance exhibited by Mtb bacilli within the infected host.^2,3^ This phenotypic heterogeneity arises primarily from the stochastic formation of a slowly replicating and metabolically distinct fraction of bacteria known as persisters, whose formation is often triggered by antibiotic treatment.^4–7^ Persisters are largely resistant to antibiotic effects and can typically only be eliminated through a combination of multiple antibiotics over an extended treatment period. The lengthy treatment duration is a major extrinsic factor that contributes to noncompliance among TB patients, which, in turn, leads to the emergence of multidrug-resistant (MDR) TB. The uncontrolled emergence and spread of MDR-TB pose significant challenge in the global TB pandemic. To combat MDR-TB, it is crucial to understand the intrinsic factors within Mtb that contribute to the development of MDR and to discover new treatments that specifically prevent the emergence of MDR-TB during chemotherapy.

Persisters are a phenotypic variant that is transiently but highly tolerant to nearly all TB antibiotics. Their formation is determined by metabolic remodeling rather than genetic changes.^8^ The formation of persisters and the accompanying antibiotic tolerance are well-known intrinsic factors that Mtb employs to survive antibiotic effects. Intriguingly, the level of antibiotic susceptibility remains unaltered when persisters regrow under antibiotic-free conditions. A substantial body of evidence indicates that persisters surviving lethal doses of TB antibiotics are responsible for a range of chronic and recurrent infections upon regrowth.^9–11^ Additionally, persisters are recognized as a source of genetic mutation-mediated MDR. These bacilli typically show resilience to high levels of DNA damage caused by the accumulation of reactive oxygen species (ROS) within the bacilli, which is induced by treatment with bactericidal antibiotics.^8,12–15^

Intensive research has focused on elucidating the molecular mechanisms behind persister biology in an effort to advance TB chemotherapeutics. Environmental cues such as nutrient starvation, redox stress, low pH, low oxygen tension, and uncured DNA damage have been studied as factors that trigger persister formation.^11,16–18^ Separate studies have revealed bacteria-specific intrinsic factors that bacteria use to adapt to these environmental stresses. One example is the toxin-antitoxin (TA) modules of *Escherichia coli*.^19,20^ Overexpression of the toxin or downregulation of the antitoxin significantly reduces growth rates and induces levels of antibiotic tolerance, a phenomenon frequently observed in the persister-rich subfraction of *E. coli*. Recently, metabolic remodeling has emerged as one of the strategies used by bacterial pathogens, including Mtb, to generate genetic mutation-free persisters and escalate antibiotic tolerance.^7,8,13,21–24^ Recent metabolomics studies have shown that Mtb phenotypic heterogeneity is attributed to the formation of a new subpopulation with altered central carbon metabolism (CCM) activity. This remodeling includes the capacity to co-catalyze multiple carbon sources, such as glycolytic and gluconeogenic carbons, to support Mtb’s full replication rate. It also involves the catabolic remodeling of cell wall glycolipids to enhance Mtb persister biology, bypassing the oxidative branch of the TCA cycle to downregulate the production of NADH. Additionally, Mtb can reciprocally induce carbon flux through the glyoxylate shunt or methylcitrate cycle and enhance the biosynthesis of succinate, followed by active secretion, to optimize membrane bioenergetics under diverse environmental stresses.^8,13,22,25^ Thus, metabolic remodeling serves as a crucial intrinsic factor employed by Mtb persisters used to survive antibiotic effects. Mtb persisters require a distinct metabolic network that provides a phenotypic advantage, enabling them to withstand antibiotic stresses and associated DNA damage.^21,22,24,26–31^

Phenotypic heterogeneity has recently garnered significant attention as an additional intrinsic factor of Mtb that promotes adaptive evolution.^32^ Expanding the bacterial subpopulation with a diverse range of metabolic activities can lead to the emergence of genotypes distinct from those of the original population. The newly generated subpopulation can serve as a source of bacilli that withstand bactericidal stresses. Recent studies have utilized mathematical simulation to model drug resistance during bacterial infection. These studies have revealed that even small increases in mutation rates considerately accelerate the frequency of emerging drug resistance, primarily due to induced phenotypic heterogeneity.^33,34^ A proof-of-concept study in yeast supported these findings by demonstrating that initial drug-resistance mutations in a small fraction resulted in higher minimal inhibitory concentration (MIC) of antibiotics and accelerated the emergence of MDR. Additionally, increased expression of efflux pumps heightened mutation rates through a previously unknown mechanism involving *mutS* and further increased phenotypic heterogeneity.^35,36^ An intriguing example of how metabolic network remodeling promotes phenotypic heterogeneity is the random emergence of persisters, often referred to as a “gambler” subpopulation.^33^ When bacterial pathogens are exposed to antibiotics, only a small fraction of the bacilli activate their DNA repair systems.^37^ Although the SOS stringent response is triggered, only a specific subset of bacilli experiences increased levels of ROS and the subsequent activation of the stress response. Since both the SOS and general stress responses are essential for mutagenic DNA break repair, persisters exhibit a significantly higher mutagenic rate. Furthermore, ample evidence has shown that adaptive metabolic network remodeling plays a crucial role in enhancing phenotypic heterogeneity, thereby providing a source of gaining evolutionary advantages through frequent DNA mutagenesis.

We have recently identified that trehalose synthase (TreS) plays a crucial role as a mediator in driving metabolic remodeling and phenotypic heterogeneity, leading to the formation of Mtb persisters capable of surviving antibiotic effects.^13,22^ Trehalose, a non-reducing glucose disaccharide, is highly abundant in Mtb. It serves both as a carbohydrate store and a core component of cell wall glycolipids, such as trehalose monomycolate (TMM) and trehalose dimycolate (TDM).^38^ Metabolomics studies conducted with Mtb bacilli collected from *in vitro* biofilm cultures revealed that a TreS-centered catalytic redirection is critical to Mtb persister biology. This redirection channels free trehalose towards the biosynthesis of intermediates in the CCM, providing an alternate source of energy and antioxidants, while diverting it away from the biosynthesis of TMM and TDM. As anticipated, a *treS*-deficient Mtb mutant (ΔtreS), which lacks the trehalose catalytic shift, exhibited heightened sensitivity to all tested TB antibiotics. These findings underscore the importance of the TreS-mediated trehalose catalytic shift and subsequent metabolic remodeling in Mtb persister formation and enhanced antibiotic tolerance. We have also recently developed TreS-specific inhibitors and confirmed their potential as adjunctive therapeutic candidates.^39^ Notably, the trehalose catalytic shift appears to be more active in DR-TB clinical isolates than in drug-sensitive (DS)-TB clinical isolates. This suggests that the trehalose catalytic shift may be a strategy employed to facilitate the emergence of drug resistance.

In this study, we demonstrated that inactivating the trehalose catalytic shift using the CRISPR*i*-dCas9 technology not only limited persister formation and phenotypic heterogeneity but also reduced rates of drug resistance against key first-line TB antibiotics, rifampicin (RIF) and isoniazid (INH). We employed mathematical modeling to show that mycobacterial bacilli enhance their chances of acquiring drug resistance by increasing trehalose catalytic shift activity and phenotypic heterogeneity. The mathematical modeling also indicated that the conversion frequency from persisters to drug-resistant mutants was nearly the same for both wildtype and ΔtreS, suggesting that the metabolic remodeling involved in inducing persister formation also plays a significant role in the emergence of drug resistance. Additionally, we identified new Mtb subfraction, termed pre-resistant bacilli, which appears before the acquisition of drug resistance. Unlike persisters, these bacilli can grow even under antibiotic stress, and their formation is largely attributed to enhanced trehalose catalytic shift activity. This phenotypic heterogeneity, resulting from the formation of persisters or pre-resistant bacilli, is a critical intrinsic factor in Mtb that contributes to the accumulation of drug resistance. Therefore, the trehalose catalytic shift represents a potential target for adjunctive therapeutic strategies, not only to better understand Mtb phenotypic heterogeneity but also to prevent the emergence of MDR-TB cases.

## Results

Recent reports indicate that trehalose acts as a growth-permissive carbon source for DR-TB clinical isolates.^13^ However, the trehalose-mediated growth was reversed when co-treated with a TreS-specific inhibitor, validamycin A (ValA).^13^ Metabolomics profiling further supported the central role of the TreS-centered trehalose catalytic shift in the metabolic networks of DR-TB clinical isolates. The catalytic conversion of trehalose into intermediates of glycolysis and the pentose phosphate pathway (PPP) suggests that DR-TB clinical isolates prefer trehalose as a substrate for the biosynthesis of CCM intermediates, rather than for the production of cell wall glycolipids like TDM. These observations led us to hypothesize that the trehalose catalytic shift not only contributes to transient antibiotic tolerance but also plays a critical role in the emergence of multidrug-resistant mutants.

### Trehalose metabolism differs in DR-TB and DS-TB clinical isolates.

**Figure 1:**
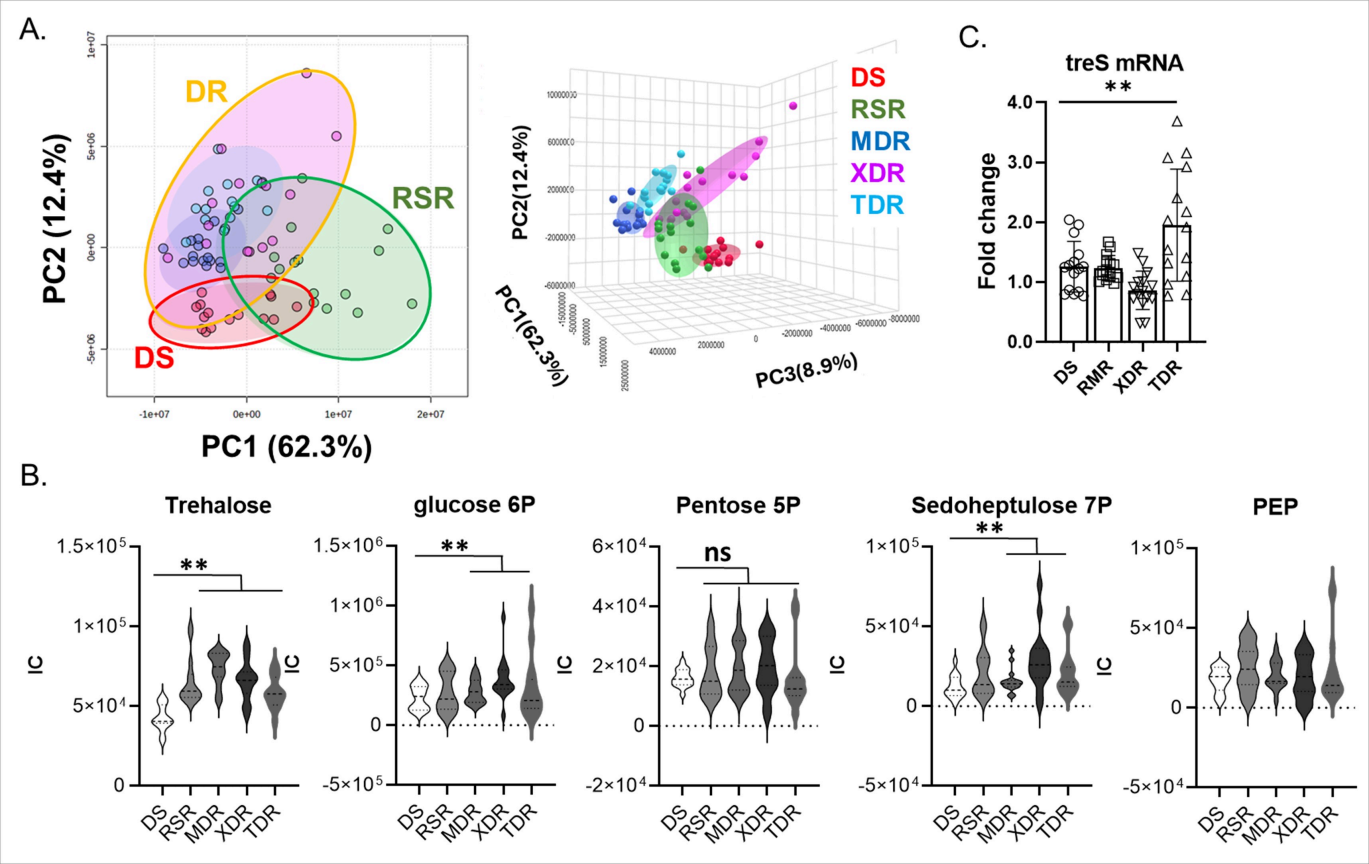
Metabolic networks of DS-TB and DR-TB clinical isolates altered by trehalose supplementation. (A) 2D (left panel) and 3D (right panel) principal component analysis (PCA) of the metabolome profiles of DS- (red), RSR- (green), MDR- (blue), XDR- (magenta), and TDR-TB (light blue) clinical isolates cultured in m7H9 containing 20 mM trehalose. (B) Targeted metabolomics analysis focusing on intermediates in trehalose metabolism, glycolysis, and the pentose phosphate pathway. PEP, phosphoenolpyruvate. Data points represent the average of 15 biological replicates ± s.e.m. **, P < 0.05; ns, not significant by Student’s t-test. (C) qRT-PCR analysis of *treS*in TB clinical isolates. **P < 0.01, as determined by Student’s t-test.

To further investigate the trehalose metabolism networks in DR-TB and DS-TB clinical isolates, we collected a total of 75 TB clinical isolates from the TB clinical isolate library at the International Tuberculosis Research Center (ITRC). This collection included 15 DS-TB, 15 rifampicin single-resistant (RSR)-TB, 15 MDR-TB, 15 extensively drug-resistant (XDR)-TB, and 15 totally drug-resistant (TDR)-TB clinical isolates (Table S1). All strains in each category were cultured in Middlebrook 7H9 liquid medium (m7H9) supplemented with sodium butyrate (SB), a permissive carbon source for all clinical isolates.^13,23^ The growth of DS-TB and DR-TB clinical isolates was enhanced by the addition of 20 mM trehalose; however, the growth of DR-TB, but not DS-TB, clinical isolates was reduced when co-treated with ValA (Fig. S1A). Although the heterogenous growth kinetics of DR-TB clinical isolates complicated appropriate statistical analyses, the specific impact of ValA on the growth of DR-TB clinical isolates in m7H9 containing trehalose clearly indicated that DR-TB clinical isolates rely more on the TreS activity to utilize exogenously supplied trehalose compared to DS-TB clinical isolates (Fig. S1A). To examine their metabolic networks, we extracted the total metabolome after culturing the isolates in m7H9 containing 20 mM trehalose. We determined the trehalose-induced metabolic networks of TB clinical isolates by monitoring approximately 200 TB metabolites and comparing their abundances in DR-TB clinical isolates with those in DS-TB clinical isolates. Using bioinformatics tools available in MetaboAnalyst (v.6.0), we identified metabolic networks unique to DR-TB clinical isolates by specifically focusing on those involved in trehalose consumption. Hierarchical clustering analysis revealed distinct metabolomics patterns between all DR-TB and DS-TB clinical isolates as depicted in the heatmap and phylogenetic tree (Fig. S1B). Principal Component Analysis (PCA) further confirmed the divergence in metabolomics patterns between the two categories of TB clinical isolates (Fig. 1A). These analyses demonstrated that the metabolic networks in DS-TB clinical isolates used to consume trehalose differed from those in MDR- and XDR-TB clinical isolates but were relatively similar to those of RSR-TB clinical isolates (Figs. 1A, S1B). We identified metabolites in DR-TB clinical isolates that were significantly altered compared to those in DS-TB clinical isolates, conducted pathway mapping, and found that trehalose metabolism, the D-alanine pathway, and the PPP were among the top-ranked pathways. Targeted metabolomics analysis indicated that trehalose abundance was significantly greater in all DR-TB clinical isolates (Fig. 1B, left panel). Furthermore, the biosynthesis of glycolysis and PPP intermediates, such as glucose 6-phosphate (Glc6P), pentose 5-phosphate (Pen5P), and sedoheptulose 7-phosphate (S7P) in DR-TB clinical isolates was either similar to or significantly greater than that in DS-TB clinical isolates (Fig. 1B, middle panels). This suggests that a substantial portion of exogenously supplied trehalose is utilized for the biosynthesis of intermediates in glycolysis and PPP in DR-TB clinical isolates.^13^ Consistent with previous findings,^23^ the level of phosphoenolpyruvate (PEP), the most downstream intermediate in glycolysis was similar across all clinical isolates (Fig. 1B, right panel). In contrast to the metabolites in upper glycolysis and PPP, those in the TCA cycle of all DR-TB clinical isolates were either unaltered or downregulated (Fig. S1C). Notably, the *treS* mRNA expression in all DR-TB clinical isolates remained unaltered, although it was slightly induced in TDR-TB clinical isolates (Fig. 1C). Collectively, these findings indicate that the metabolic networks involved in trehalose consumption are organized differently between DS-TB and DR-TB clinical isolates, with regulation occurring independently of transcriptional changes.

### TreS-deficient M. smegmatis phenocopied Mtb mutants that lack trehalose catalytic shift.

**Figure 2:**
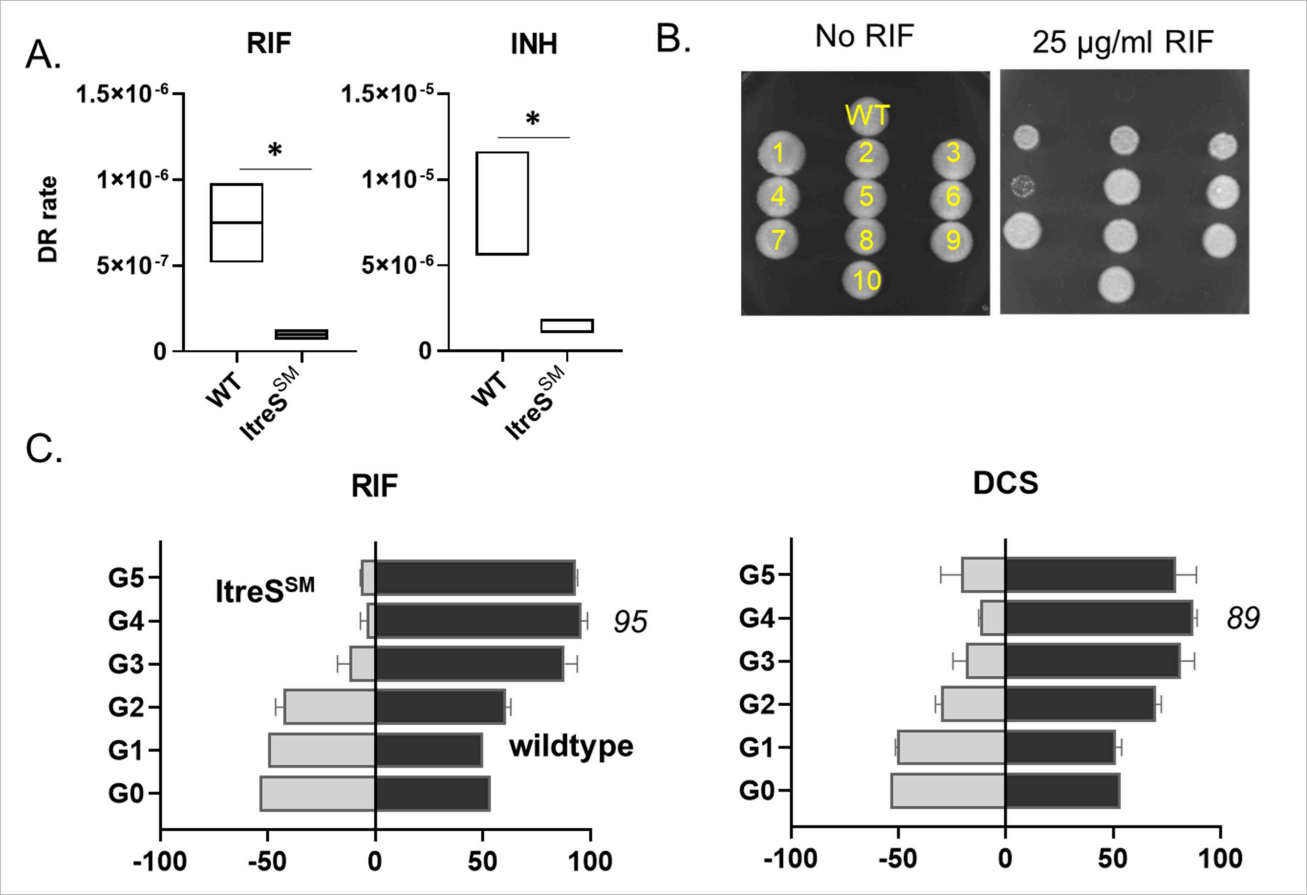
Trehalose catalytic shift as an adaptive strategy for the emergence of drug-resistant mycobacterial mutants. (A) The rates at which the indicated mycobacterial strains acquired resistance to RIF (left panel) or INH (right panel) per generation were measured using the classical fluctuation assay. All values represent the average of 25 biological replicates ± s.e.m. *, P < 0.01 as determined by Student’s t test. (B) Colony formation in a spot assay. Ten colonies from RIF-resistant bacilli obtained from (A, left panel) were spotted on m7H10 containing either no RIF or 25 μg/mL RIF. WT, naïve drug-sensitive *M. smegmatis* bacilli. (C) Coculture of wildtype *M. smegmatis* expressing green fluorescence protein (GFP) and ItreS^SM^ expressing red fluorescence protein (RFP) was subjected to intermittent exposure to RIF over a total of 5 cycles, referred to as G0-G5 cultures. The relative enrichment of wildtype and ItreS^SM^ in G0 to G5 cultures was calculated via flow cytometry and is represented as a percentage. Gray bar represents ItreS^SM^; black bar represents wildtype.

To study the role of trehalose catalytic shift in the development of drug resistance in mycobacterial bacilli, we employed the recently developed CRISPR*i*-dCas9 technique to inducibly deplete *treS* gene expression in *M. smegmatis* (Fig. S2A).^40,41^ The CRISPR*i treS* mutants of *M. smegmatis* (termed ItreS^SM^) were cultured in the mid-log phase, and *treS* knockdown was induced using various concentrations of anhydrotetracycline (ATc). The efficacy of *treS* mRNA knockdown was assessed by qRT-PCR with treatment at 200 ng/mL ATc resulting in approximately 90% suppression (Fig. 2B, left panel). We also created IotsA^SM^ to knockdown *otsA*, a gene responsible for encoding trehalose 6P synthase involved in Mtb trehalose metabolism, but not associated with the trehalose catalytic shift. Similar to the observation in *treS*-deficient Mtb (ΔtreS),^13^ ItreS^SM^ produced persister-like bacilli within the *in vitro* biofilm culture (referred to as biofilm-persisters) at a significantly lower level than wildtype following ATc treatment. In contrast, both IotsA^SM^ and wildtype were able to form mature biofilm-persisters, despite showing no discernible growth defects in Sauton media (Fig. S2C, D). ItreS^SM^ without ATc was included as a complement condition and exhibited the ability to form biofilm-persisters at a level similar to that of wildtype (Fig. S2D). Targeted metabolomics of ItreS^SM^ revealed that the inability to form intact biofilm-persisters was primarily due to impaired trehalose catalytic shift, which affected the trehalose-mediated carbon flux through glycolysis and the PPP (e.g., Glc6P, glyceraldehyde 3P, and S7P) (Fig. S2E). Recently, the depletion of both PEP abundance and the PEP/pyruvate ratio has been identified as a metabolic strategy employed by Mtb to induce persister formation, slow its replication rate, and enhance antibiotic tolerance.^23^ Notably, ItreS^SM^ showed accumulated PEP as compared to that of wildtype (Fig. S2E). As a result, ItreS^SM^ exhibited increased susceptibility to antibiotics, such as RIF, INH, and BDQ compared to wildtype or IotsA^SM^ (Fig. S2F), similar to the phenotype observed in ΔtreS Mtb.^13^ These findings collectively indicate that ItreS^SM^ phenocopies ΔtreS Mtb.

### The trehalose catalytic shift is an adaptive strategy to emerge drug-resistant mycobacterial mutants.

If Mtb persisters survive antibiotic-induced bactericidal oxidative stresses, such as ROS which are known DNA mutagen, their prolonged survival may be linked to the development of drug-resistant mutations. The metabolic strategies employed by Mtb persisters during this stage are directly or indirectly involved in the emergence of drug resistance.^42,43^ To examine whether the trehalose catalytic shift is a strategy functionally associated with the emergence of drug-resistant mutants, we employed a classical Luria-Delbrück fluctuation assay to determine the rates of emerging spontaneous drug-resistant mutants in both wildtype and ItreS^SM^.^44,45^ We found that the drug-resistance rates of wildtype against RIF ranged from 5.1 X 10^− 7^ to 1 X 10^− 6^ mutations per generation (Fig. 2A, left panel). The drug-resistance rates of ItreS^SM^ without ATc were comparable to those of wildtype. RIF-resistant colonies were confirmed by spotting them on m7H10 containing high concentrations of RIF, up to 100 μg/mL (Figs. 2B and S3A). The fluctuation assay and the spot assay indicated that the mean rate of RIF resistance in wildtype was approximately 6.6-fold greater than in ItreS^SM^. Additionally, we determined the INH-resistance rates of wildtype, which ranged from 1.1 X 10^− 5^ to 5.5 X 10^− 6^ mutations per generation, while the rates for ItreS^SM^ ranged from 1.8 X 10^− 6^ to 1.0 X 10^− 6^ mutations per generation. The wildtype exhibited INH resistance development at levels approximately 5.4-fold greater than that of ItreS^SM^ (Fig. 2A, right panel). These findings suggest a functional link between the trehalose catalytic shift and the frequency of drug resistance development in mycobacterial bacilli against first-line TB antibiotics, irrespective of the modes-of-action.

We also performed a co-culture competition assay using wildtype expressing green fluorescent protein (GFP) and ItreS^SM^ expressing red fluorescent protein (RFP) (Fig. S3B). With these two strains, we measured relative viability following cyclic exposure to bactericidal concentrations of RIF or D-cycloserine (DCS), with intermittent washing with antibiotic-free PBS, and established G1 to G5 subcultures (Fig. S3B). Flow cytometry analysis was utilized to determine the relative abundance of wildtype::GFP and ItreS^SM^::RFP within the G0 to G5 subcultures (Fig. 2C). The iterative cycle of treatment with RIF or DCS, followed by regrowth in antibiotic-free m7H9, led to a gradual accumulation of wildtype bacilli within the subcultures. In the G4 and G5 subcultures, GFP intensity became saturated but never reached 100% (Fig. 2C). This finding indicates that the G4 and G5 subcultures may contain drug-resistant bacilli from both wildtype and ItreS^SM^. Indeed, the spot assay showed that G3 subculture was the first generation to exhibit the drug-resistant phenotype, and the lag phase period during the regrowth kinetics of the G4 and G5 subcultures was nearly identical to that of naïve bacilli (Fig. S3C, D). These findings indicate that the trehalose catalytic shift represents an intrinsic strategy of Mtb that is functionally associated with the fitness cost required for natural selection and a regrowth advantage in the face of intermittent antibiotic stresses. To further support these findings, we conducted a fluctuation assay using *M. smegmatis* overexpressing *treS* (pTreS) and found that the extracopy of *treS* conferred mycobacterial bacilli resistance to RIF at levels approximately 2.0-fold greater than those of wildtype (Fig. S3E).

### DR mutants are metabolically heterogenous by forming bacilli harboring greater trehalose catalytic shift activity.

**Figure 3:**
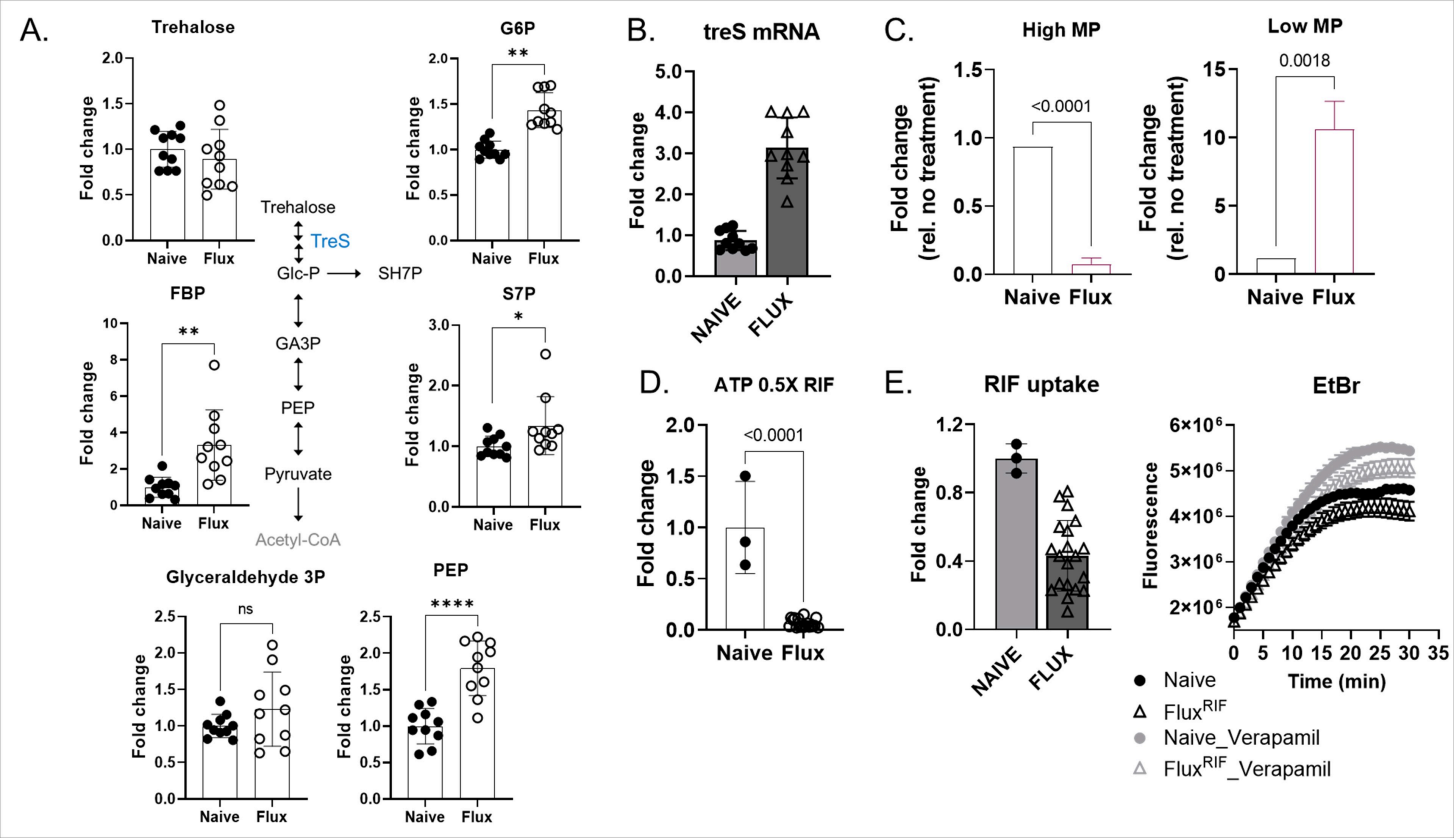
Biochemical and metabolic properties of RIF-resistant *M. smegmatis* (Flux^RIF^) bacilli. (A) Targeted metabolomics profiles. The analysis focused on intermediates in trehalose metabolism, glycolysis, and the pentose phosphate pathway in naïve *M. smegmatis* bacilli and ten selected Flux^RIF^ bacilli. (B) Fold change of *treS* mRNA expression of naïve *M. smegmatis* and Flux^RIF^ bacilli under RIF treatment conditions relative to untreated conditions. (C) Membrane potential (ΔΨm) of naïve *M. smegmatis* and Flux^RIF^ bacilli was monitored after treatment with 30 μg/mL RIF by flow cytometry. The percentage of bacilli exhibiting high and low ΔΨm of each strain was calculated relative to the untreated condition. (D) Intrabacterial ATP concentration in Flux^RIF^ bacilli was measured, and its abundance relative to naïve bacilli is represented as a fold change. (E) Relative RIF permeability and EtBr permeability kinetics of naïve *M. smegmatis* or Flux^RIF^ bacilli were assessed. All values represent the average of biological replicates ± s.e.m. *, P < 0.05; **, P<0.01; ****, P<0.001; ns, not significant by Student’s t-test.

Using the fluctuation assay and RIF spot assay, we isolated 10 RIF-resistant *M. smegmatis* colonies, designating them as Flux^RIF^ #1-#10 (Figs. 2A, B, and S3A). Consistent with the growth kinetics of previously reported DR-TB clinical isolates,^13^ the growth patterns of all Flux^RIF^ and naïve bacilli were nearly identical in antibiotic-free m7H9 (Fig. S4A). However, while naïve bacilli were unable to form colonies on m7H10 containing RIF concentrations of 25 μg/mL or higher, all Flux^RIF^ bacilli successfully grew on the plates (Figs. 2B and S3A). Notably, Flux^RIF^ #1 and #2 bacilli carried an L_452_P mutation in the RIF-resistance determining region (RRDR),^46^ a mutation well-known to be associated with RIF resistance in many DR-TB clinical isolates.^47,48^ In contrast, Flux^RIF^ #3-#10 developed RIF resistance without any mutations in the RRDR region. To investigate the role of the trehalose catalytic shift in the observed drug-resistant phenotype of the Flux^RIF^ bacilli, we monitored their growth kinetics after supplementing with 20 mM trehalose. The addition of trehalose enhanced the growth rates of both groups of bacilli. Since ValA has minimal impact on *M. smegmatis* TreS activity, we employed the CRISPR*i*-dCas9 technique to deactivate *treS* in the Flux^RIF^ bacilli. We found that suppression of *treS* partially hindered the trehalose-induced growth of Flux^RIF^ bacilli, whereas it had little effect on the growth of naïve bacilli (Fig. S4A). This suggests a more pronounced TreS-centered trehalose catalytic shift activity in Flux^RIF^ bacilli compared to naïve bacilli. Our conclusion was further supported by metabolomics profiling, which revealed that the levels of Glc6P, fructose 1,6-bisphosphate (FBP), and S7P were significantly higher in Flux^RIF^ bacilli than in naïve bacilli, even though both strains exhibited similar levels of trehalose abundance (Fig. 3A). In contrast to the glycolysis and PPP intermediates, there were no noticeable changes in the levels of TCA cycle intermediates (Fig. S4B). Therefore, we conclude that the catalytic activities responsible for utilizing exogenous trehalose to biosynthesize glycolysis and PPP intermediates are considerably higher in Flux^RIF^ bacilli, consistent with observations from DR-TB clinical isolates (Fig. 1B).^13^ In addition, we observed that Flux^RIF^ bacilli maintained high levels of PEP despite their antibiotic tolerance, likely because they continued to replicate even in the presence of RIF (Figs. 3A and S3D).^23^ Consistent with the metabolomics profile and drug-resistant phenotype, Flux^RIF^ bacilli exhibited higher expression levels of *treS* mRNA compared to naïve bacilli, with expression levels particularly elevated in the RRDR mutation-free Flux^RIF^ #3-#10 bacilli (Figs. 3B and S4C). Moreover, Flux^RIF^ bacilli contained a larger subfraction with lower membrane potential (ΔΨm) and lower ATP levels than naïve bacilli, whose bioenergetic states resembled those of Mtb persisters (Fig. 3C, D).^13,23^ As a result, RIF antibiotic penetration into Flux^RIF^ bacilli occurred at significantly reduced levels compared to naïve bacilli, a finding further supported by the EtBr permeability assay (Fig. 3E). Taken together, these observations indicate that Flux^RIF^ bacilli exhibit increased metabolic heterogeneity by expanding the population with a greater trehalose catalytic shift and lower bioenergetic activities. This metabolic heterogeneity may contribute to the initiation of persister formation, antibiotic tolerance, and the development of drug resistance.

### The trehalose catalytic shift confers mycobacterial cells with greater metabolic heterogeneity.

**Figure 4:**
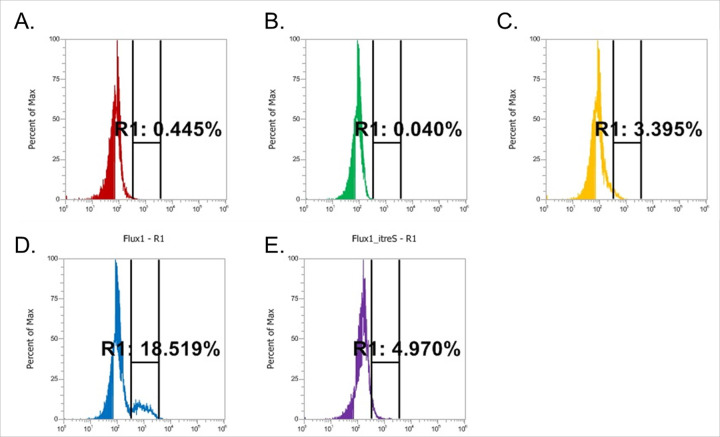
Enhanced metabolic heterogeneity of mycobacterial cells due to trehalose catalytic shift activity. RMR-tre fluorescence dye labeling pattern are shown for (A) wildtype *M. smegmatis*, (B) ItreS^SM^, and (C) pTreS^SM^ (*M. smegmatis* overexpressing TreS) following treatment with a sublethal dose of RIF. Bacilli located in the R1 area are defined as the RMR-tre^high^ population. Additionally, the RMR-tre fluorescence dye labeling patterns for (D) a selected Flux^RIF^ bacillus and (E) its CRISPR*i treS* knockdown strain after ATc treatment was depicted.

Increasing metabolic heterogeneity within an isogenic population is a well-known strategy for enhancing the generation of persisters and drug-resistant mutants.^49–51^ Recent studies have demonstrated that DR-TB clinical isolates exhibit lower TDM abundance in their cell wall due to increased trehalose catalytic shift activity.^13,23,52^ To define a functional connection between the trehalose catalytic shift of Flux^RIF^ bacilli and their ability to enhance metabolic heterogeneity, we utilized previously reported Red Molecular Rotor-trehalose (RMR-tre), a fluorogenic dye that specifically labels mycobacterial cell wall glycolipids containing trehalose as a carbohydrate core, such as TDM.^53^ The labeling intensity of RMR-tre in naïve bacilli during mid-log phase gradually decreased with the addition of increasing doses of free trehalose,^53^ suggesting that RMR-tre serves as a substrate for Ag85, an enzyme involved in TDM biosynthesis, at a level comparable to free trehalose.^54–56^ We quantified the intensity of RMR-tre labeling using FACS before and after treatment with sublethal doses of RIF. As expected, the RMR-tre labeling pattern of naïve bacilli was relatively homogenous before antibiotic treatment. However, it became heterogenous after RIF treatment, as evidenced by an increase in the subfraction of RMR-tre^high^ bacilli. This phenomenon likely occurs because mycobacterial bacilli with induced trehalose catalytic shift activity preferentially consume preexisting trehalose as a substrate for CCM intermediates, resulting in a greater level of RMR-tre incorporation compared to endogenous trehalose. Notably, these RMR-tre^high^ bacilli were absent in ΔtreS Mtb (Fig. S5A). To further investigate the extent to which the trehalose catalytic shift contributes to the formation of the RMR-tre^high^ subfraction and the associated metabolic heterogeneity, we repeated the RMR-tre labeling assay using pTreS^SM^, *M. smegmatis* overexpressing *treS*, and ItreS^SM^. Our observations revealed that the RMR-tre^high^ subfraction substantially overlapped with that of pTreS^SM^, whereas it was absent in ItreS^SM^, similar to what was observed in ΔtreS Mtb (Figs. 4A–C, S5A). This finding underscores the functional essentiality of the trehalose catalytic shift in promoting metabolic heterogeneity in response to bactericidal antibiotics. Interestingly, the fraction of RMR-tre^high^ bacilli was significantly larger in Flux^RIF^ bacilli compared to naïve DS-bacilli, even prior to antibiotic treatment (Figs. 4D and S5B, C). To determine whether the RMR-tre^high^ subfraction in Flux^RIF^ bacilli primarily consists of a viable population following treatment with bactericidal antibiotics, we tracked changes in the abundance of RMR-tre^high^ and RMR-tre^low^ subfractions after exposure to bactericidal doses of RIF. We observed a profound decrease in the RMR-tre^low^ subfraction, with the RMR-tre^high^ subfraction becoming predominant (Fig. S5D). This suggests that the metabolic heterogeneity induced by the formation of the RMR-tre^high^ subfraction is largely attributed to an enhanced trehalose catalytic shift, which is functionally related to antibiotic tolerance and the accumulation of drug-resistant mutations. Further intriguingly, this phenomenon was more pronounced in Flux^RIF^ #3-#10 bacilli than in Flux^RIF^ #1 and #2 bacilli. Flux^RIF^ #1 and #2 bacilli, which harbored the L_452_P mutation in the RRDR, maintained the RMR-tre^low^ subfraction as a dominant population even after antibiotic treatment, although there was a slight reduction in its abundance (Fig. S5D). This may occur because Flux^RIF^ #3-#10 bacilli exhibit RIF resistance due to a larger fraction harboring high trehalose catalytic shift activity. In contrast, the RIF resistance in Flux^RIF^ #1 and #2 bacilli is likely mediated by mutations in the RIF target gene. Treatment with RIF rendered all Flux^RIF^ #3-#10 bacilli relatively more homogenous, either by inducing the trehalose catalytic shift in the RMR-tre^low^ subfraction or by specifically killing the less drug-tolerant RMR-tre^low^ subfraction (Fig. S5D). This indicates that RMR-tre^high^ bacilli may represent a significant source of viable bacilli following treatment with bactericidal antibiotics. Overall, the trehalose catalytic shift is an intrinsic factor of Mtb that elevates metabolic heterogeneity and enhances its ability to survive longer under antibiotic pressure by generating the RMR-tre^high^ subfraction, which readily contributes to the formation of persisters and pre-resistant bacilli.

### The trehalose catalytic shift is necessary to elevate drug resistance frequency by increasing the persister subfraction.

**Figure 5:**
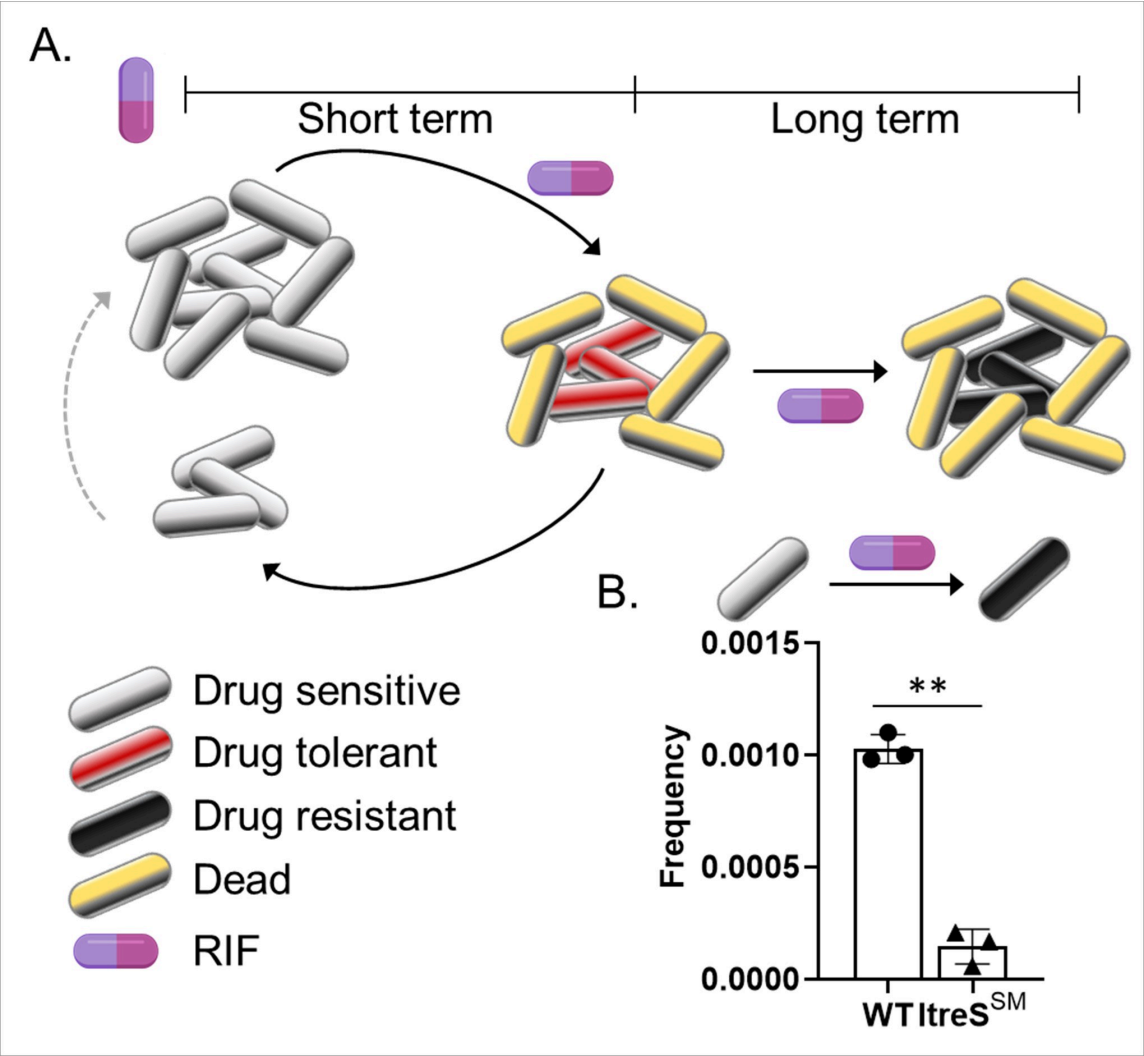
Mathematical modeling of the role of trehalose catalytic shift in the emergence of drug tolerance and drug resistance in Mtb. (A) The schematic diagram illustrates the phenotypic transitioning model, where drug-sensitive mycobacterial bacilli (gray) evolve into drug-resistant bacilli (black) via repetitively forming drug-tolerant bacilli (red). In this model, mycobacterial bacilli can reversibly switch between drug-sensitive and drug-tolerant states. Once a bacillus becomes drug-tolerant, it remains in that state for multiple generations before reverting to a drug-sensitive state. Following prolonged antibiotic exposure, each drug-tolerant bacillus irreversibly achieves permanent drug resistance. To assess the impact of the trehalose-catalytic shift on the frequency of each step in phenotypic transitioning, wildtype *M. smegmatis* and ItreS^SM^ were utilized in a fluctuation assay as depicted in Fig. S6A. (B) The rates forming drug-resistant mutants in wildtype and ItreS^SM^ against RIF were calculated using the classical fluctuation assay and the Lea-Coulson method (m/Nt where m is the number of resistant colonies and Nt is the total input).

Pathogenic bacteria can transiently acquire a drug-tolerant phenotype through a non-genetic mechanism by forming persisters. They subsequently regrow as a species when the effects of antibiotics diminish. This cycle repeats until drug-resistant mutants emerge (Fig. 5A). The phenotypic reversibility between drug-sensitive bacilli and drug-tolerant persisters occurs when antibiotic priming is intermittent. Continuous antibiotic pressure, however, leads to the accumulation of drug-resistant mutations.^57,58^

We have employed mathematical modeling to create analytical formulas that predict the impact of a trehalose-catalytic shift on the kinetics of reversibility and the observed clone-to-clone fluctuations within the population that survives antibiotic stresses. This surviving population ultimately serves as a reservoir for drug-resistant bacilli (Fig. 5A).^59,60^ To capture the emergence of drug-tolerant persisters during population growth, we have developed a model in which single bacilli reversibly switch between drug-sensitive and drug-tolerant states.^61^ Once a bacillus becomes drug-tolerant, it remains in that state for multiple generations before reverting to a drug-sensitive state (Fig. 5A). Our previous work has modeled such a reversible switching in the context of a fluctuation assay, allowing us to analytically predict the expected statistical variation in the number of drug-tolerant bacilli across colonies derived from a single bacillus. Our analysis of the fluctuation assay data using this reversible switching model indicates that ItreS^SM^ persisters are more unstable than wildtype persisters, reverting to a drug-sensitive state more quickly. Additionally, our findings reveals that the observed lower number of resistant colonies in the ItreS^SM^ compared to wildtype (Fig. 5B) is predominantly due to a six-fold lower rate of persister formation in ItreS^SM^ (see Mathematical Modeling in the Method section). The emergence of drug-resistant bacilli is known to be facilitated by an increased number of persisters.^8,50,62–66^ Drug-resistant mutants exhibit metabolic similarities to Mtb persisters (Figs. 3, 4, S4, and S5). Thus, we conclude that mycobacterial bacilli evolve into drug-resistant mutants through the repetitive formation of drug-tolerant persisters and pre-resistant bacilli. The trehalose catalytic shift serves as a strategy to enhance the subfraction of persisters and pre-resistant bacilli under high levels of ROS damage, thereby facilitating the emergence of drug-resistant mutants.

### RIF-resistant mycobacterial cells are also resistant to INH and BDQ.

**Figure 6:**
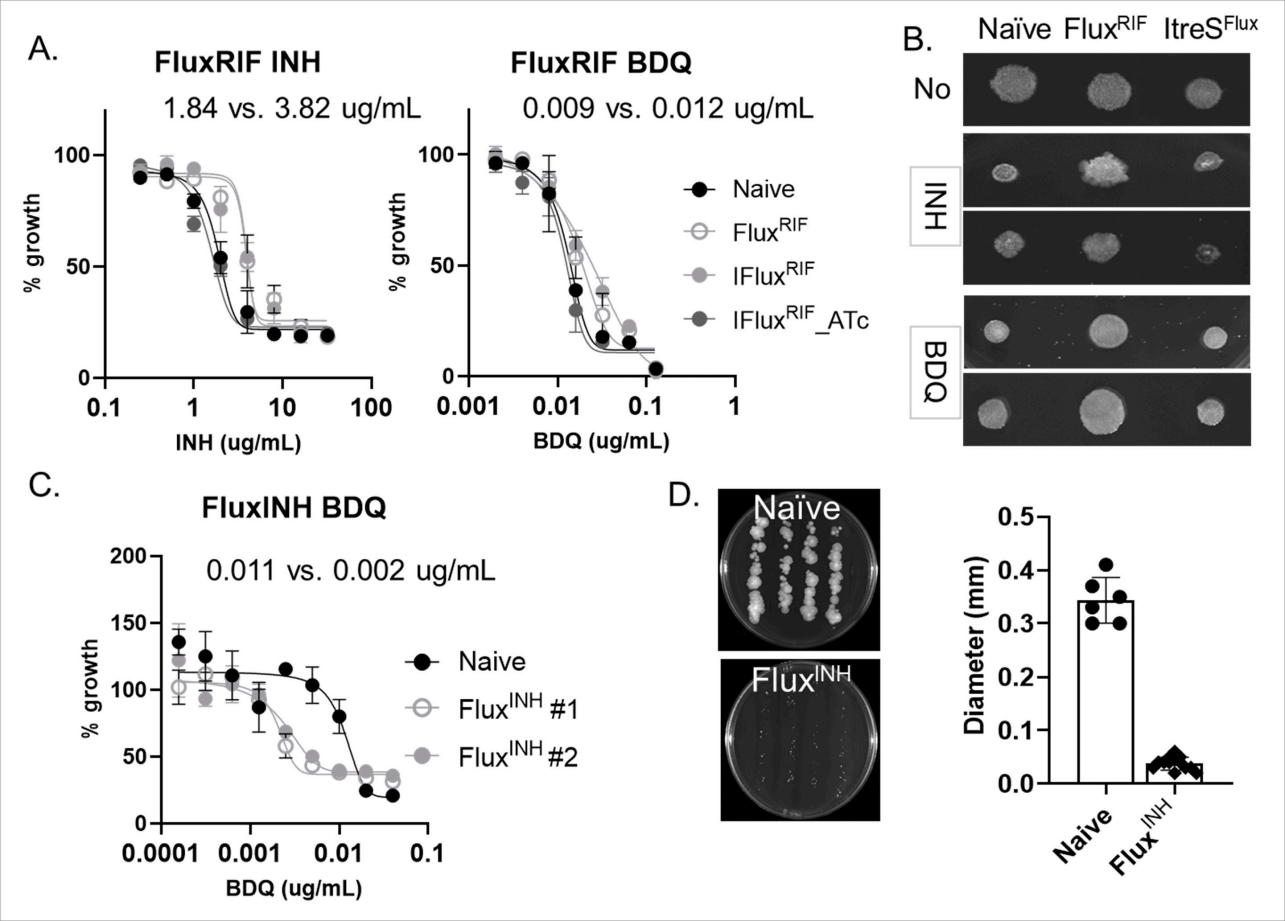
The role of trehalose catalytic shift in the emergence of antibiotic cross-resistance. (A) IC_50_ values for INH (left panel) and BDQ (right panel) were assessed in naïve *M. smegmatis*, Flux^RIF^, and ItreS^Flux^ bacilli, both with and without ATc treatment. (B) A spot assay was performed on m7H10 containing 10X MIC INH or BDQ, using naïve *M. smegmatis*, Flux^RIF^, and ItreS^Flux^ bacilli. (C) IC50 values for BDQ were determined for naïve *M. smegmatis* and two selected Flux^INH^ bacilli. (D) A spot assay on m7H10 containing 10X MIC of BDQ (left panel) was conducted with naïve *M. smegmatis* and Flux^INH^ bacilli. The right panel displayed the average diameters and the standard deviation of colonies formed on m7H10 containing 10X MIC BDQ.

Reports from TB clinical isolates at Taiwan Medical Center indicate that 94.6% of RIF-resistant strains were also resistant to INH while only 0.5% were mono-resistant to RIF.^67^ A similar pattern was observed in the retrospective TB case studies conducted in New York City between 2010 and 2021.^68^ These findings suggest that RIF resistance may serve as a predictive biomarker for MDR-TB. Therefore, we hypothesized that RIF-resistant strains could possess a metabolic advantage that confers greater tolerance to second antibiotics, such as INH, even without prior exposure to these antibiotics. RMR-tre labeling patterns indicate that Flux^RIF^ bacilli contain a high abundance of RMR-tre^high^ subfraction (Figs. 4D and S5B, C). To investigate this hypothesis, we conducted a minimum inhibitory concentration (MIC) shift assay using selected Flux^RIF^ bacilli and their CRISPR*i treS* mutant, referred to as ItreS^Flux^, comparing their antibiotic sensitivity to that of naïve bacilli. Flux^RIF^ bacilli exhibited significantly higher tolerance to INH, with MIC values of approximately 3.82 μg/mL, compared to around 1.84 μg/mL for naïve bacilli. However, this INH tolerance diminished in ItreS^Flux^ after treatment with ATc, resulting in an MIC value at around 1.49 μg/mL (Fig. 6A, left panel). This finding was not observed in ItreS^Flux^ without treatment with ATc. This suggests that Flux^RIF^ bacilli are better equipped to withstand the effects of INH, likely due to their greater abundance of the RMR-tre^high^ subfraction (Figs. 4D and S5D). A spot assay performed on m7H10 containing bactericidal doses of INH corroborated the results of the MIC shift assay (Figs. 6B and S7A). Additionally, Flux^RIF^ bacilli demonstrated higher tolerance to BDQ as well, underscoring the significant role of the trehalose catalytic shift in cross-resistance to various TB antibiotics (Fig. 6A, right panel). This is further supported by the fact that the ITRC TB clinical isolate library includes only 15 RSR-TB clinical isolates (less than 1%) among a collection of over 1,500 clinical isolates. Surprisingly, the inverse relationship of cross-resistance was not clearly detected. INH-resistant bacilli (referred to as Flux^INH^), obtained from the fluctional assay (Fig. 2A, right panel), were collected and tested for their antibiotic sensitivity against RIF. The MIC shift assay and colony size measurement conducted on two randomly selected Flux^INH^ bacilli revealed that they were significantly more sensitive to RIF compared to naïve bacilli (Fig. 6C, D), likely due to their increased RIF permeability (Fig. S7B, upper panel). This altered membrane permeability was further supported by the EtBr permeability assay (Fig. S7B, lower panel). INH is a prodrug that requires structural activation through the formation of an NAD^+^ adduct to exhibit its antimicrobial activity.^69^ As shown in Fig. 3, Flux^RIF^ bacilli demonstrated distinct metabolic networks compared to naïve bacilli, primarily attributed to a higher trehalose catalytic shift and concurrently lower membrane bioenergetics, characterized by reduced levels of NAD^+^, ΔΨm, and ATP (Fig. 3C, D). This metabolic state likely influences the formation of INH-NAD adducts. The increased cross-resistance of Flux^RIF^ bacilli to INH or BDQ was significantly downregulated by inhibiting *treS* using CRISPR*i*-dCas9 technique (Fig. 6A). This supports the hypothesis that the trehalose catalytic shift contributes to the emergence of MDR-TB cases.

### The trehalose catalytic shift enables HN878 W-Beijing strain to acquire a high frequency of multidrug resistance.

**Figure 7:**
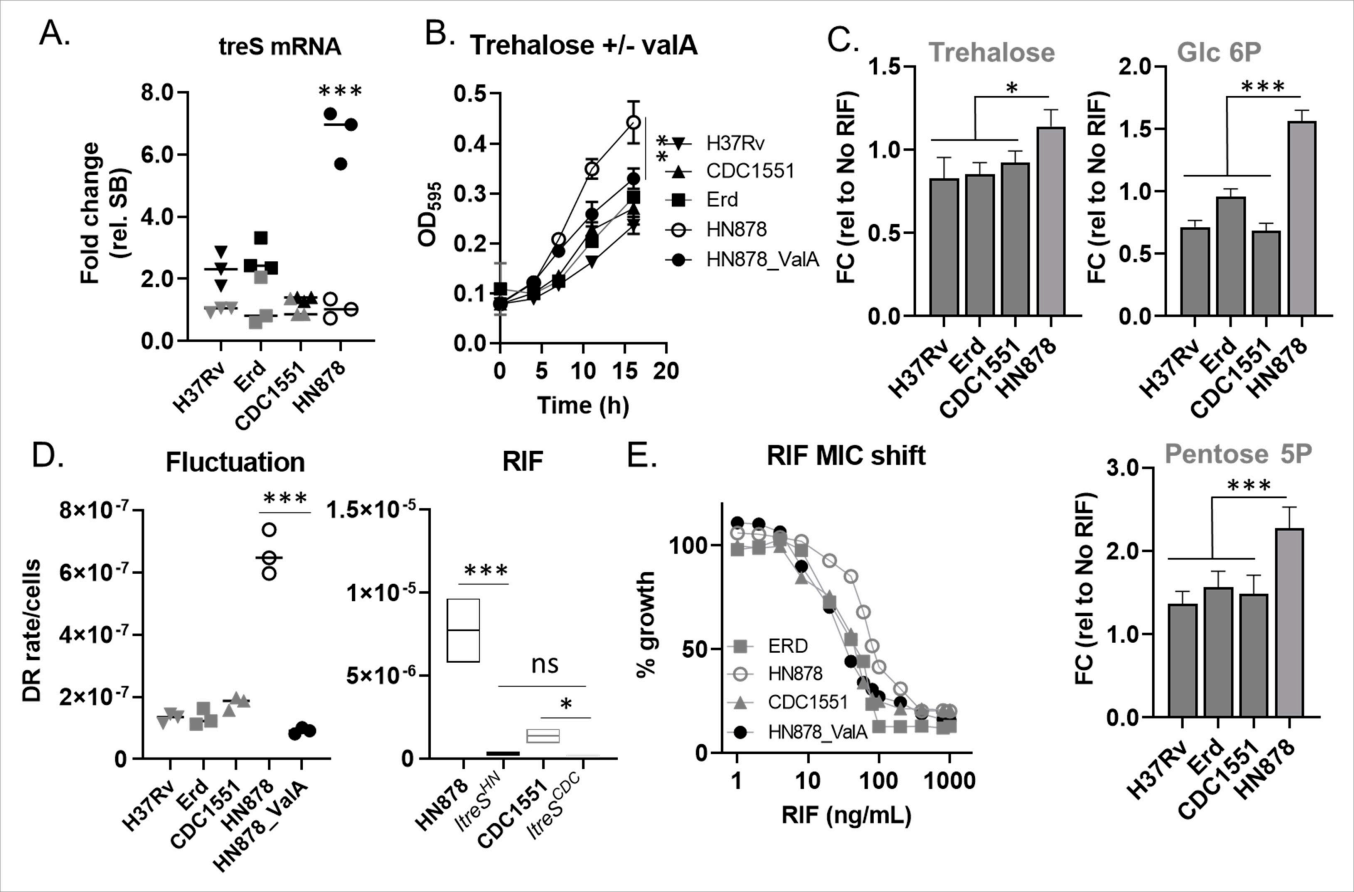
Characterization of HN878’s trehalose catalytic shift activity. (A) The expression of *treS* mRNA in HN878 and lineage 4 strains (e.g., H37Rv, Erdman, and CDC1551) was measured before and after treatment with RIF. The closed black circles represent the changes in mRNA levels following RIF treatment, with fold changes depicted relative to untreated counterparts. (B) Growth kinetics of HN878 and lineage 4 strains on m7H10 containing trehalose as the sole carbon source were shown. The impact of ValA on the growth of HN878 is also illustrated. All values represent the average of triplicates ± s.e.m. **, P<0.01 by Student’s t-test. (C) RIF treatment induced changes in the levels of trehalose, glucose 6P, and pentose 5P in HN878 and lineage 4 strains, relative to the no-treatment condition. (D) The effects of ValA (left panel) or CRISPR*i*-mediated *treS* inactivation (right panel) on the rates at which HN878 and lineage 4 clinical strains acquired RIF resistance per generation were measured using the classical fluctuation assay. (E) The impact of ValA on IC_50_ values of RIF against the indicated Mtb clinical strains is shown: HN878 (~62 ng/mL), ERD (~31 ng/mL), CDC1551 (~22 ng/mL) and HN878 treated with ValA (~22 ng/mL). All values are the average of biological triplicates ± s.e.m. *, P<0.05, **, P<0.01; ***, P<0.001; ns, not significant (by Student’s t-test).

Clinical Mtb strains are categorized into phylogeographic lineages 1 through 7, each exhibiting varying capacities for acquiring MDR mutations.^45,70^ Lineage 2 strains, including the HN878 W-Beijing strain (HN878), have been associated with a heightened risk of MDR-TB emergence on a global scale. Our findings suggest that the trehalose catalytic shift in Mtb contributes to an increased frequency of MDR mutations by promoting the formation of persisters and cross-resistance to multiple antibiotics (Figs. 4, 5, and 6), Therefore, we hypothesize that elevated trehalose catalytic shift activity in HN878 plays a key role in its propensity to accumulate MDR mutations more frequently than other lineage strains. To investigate this hypothesis, we examined TreS activity in HN878 following exposure to sublethal doses of RIF. The expression of *treS* mRNA in HN878, as well as in lineage 4 strains such as Erdman or CDC1551, was notably upregulated in response to RIF treatment. Interestingly, the induction of *treS* mRNA in HN878 increased by approximately 7.3-fold compared to the untreated controls, which was significantly higher than the 2 to 3-fold increase observed in lineage 4 strains (Fig. 7A). We also found that HN878 exhibited faster growth rates than the lineage 4 strains in m7H9 containing trehalose as the sole carbon source (Fig. 7B). Co-treatment with ValA restored trehalose-mediated growth to levels comparable to those of lineage 4 strains, suggesting that trehalose may serve as a more favorable carbon source for HN878, likely due to its higher TreS activity (Fig. 7B). The catalytic activities involved in converting consumed trehalose into glycolysis and PPP intermediates such as Glc6P, Pen5P, and S7P, were significantly higher in HN878 than in lineage 4 strains, further supporting our hypothesis (Fig. 7C). Collectively, these findings suggest that HN878 undergoes a greater trehalose catalytic shift compared to lineage 4 strains, leading to the development of MDR mutations more frequently in HN878 than in other lineage strains.

To further validate the functional importance of the trehalose catalytic shift in HN878 for the emergence of drug-resistant mutants, we conducted a fluctuation assay using HN878 and lineage 4 strains, both with and without ValA, as well as the CRISPR*i treS* mutant of HN878 (ItreS^HN^), CDC1551 (ItreS^CDC^), or Erdman (ItreS^Erd^) (Figs. S2A and S8A). Consistent with previous literature,^45^ we observed that HN878 exhibited approximately a 5.0-fold higher frequency of developing RIF resistance compared to lineage 4 strains (Fig. 7D, left panel). Treatment with ValA significantly reduced the mutation rates to levels comparable to those of lineage 4 strains (Fig. 7D, left panel). Similar results were observed with the CRISPR*i treS* mutants, where the rates of drug-resistant mutations for ItreS^HN^ and ItreS^CDC^ against RIF became comparable (Fig. 7D, right panel). Furthermore, HN878 showed a significantly higher MIC of RIF (~ 0.06 μg/mL) due to its enhanced trehalose catalytic shift activity, compared to lineage 4 strains (~ 0.03 μg/mL). However, when co-treated with ValA or in ItreS^HN^, the MIC value decreased to approximately 0.02 μg/mL (Figs. 7E and S8B, C). To establish a link between the enhanced trehalose catalytic shift and its metabolic heterogeneity, as well as persister formation and drug tolerance in HN878, we utilized the most probable number (MPN) assay. This recently innovated method monitors the abundance of total persisters, which includes traditional persisters and differentially detectable (DD) bacilli under RIF treatment and nutrient-starved conditions.^71,72^ We found that the frequency of persister formation in HN878 was the highest among all clinical strains tested in this study (Fig. S8D). The reduction rate after co-treatment with ValA (Fig. S8D, left panel) or using ItreS^HN^ (Fig. S8D, right panel) was the largest, suggesting that the high frequency of MDR development in HN878 is largely attributed to its greater trehalose catalytic shift activity and the resulting persister formation. According to our mathematical modeling results (Fig. 5), the frequent emergence of MDR-TB cases linked to infections with HN878 is primarily due to elevated levels its trehalose catalytic shift activity and persister formation. Thus, the trehalose catalytic shift represents a promising target for novel adjunctive therapeutics aimed at preventing the emergence of MDR-TB.

## Discussion

Persister formation is a widespread adaptive strategy among bacterial pathogens including Mtb, allowing them to survive the effects of antibiotics for extended periods without developing genetic mutations that confer drug resistance.^8,73^ The pathogenic cycle of TB includes an intractable latent infection stage, during which Mtb bacilli often enter a persister state. In this state, they can opportunistically recur, increasing the bacterial burden and serving as a source for potential genetic mutations that lead drug resistance.^33,74,75^ Compared to heritable drug resistance, the biology of Mtb persisters is still in the early stages of investigation. The present study indicates that mycobacterial persisters are indeed an adaptive method that plays a crucial role in the optimal pathogenic cycle of TB. Persister formation is triggered by metabolic remodeling, such as trehalose catalytic shift, and is directly or indirectly linked to the evolutionary traits that give rise to drug-resistant mutants.^8,11,76–79^ In addition to serving as a source of intermittent antibiotic tolerance and opportunistic recurrence, our findings support the notion that Mtb persisters act as a reservoir for the emergence of multidrug resistance and contribute to the spread of MDR-TB.^52,63,64,75,80–82^ Notably, the accumulation of ROS resulting from antibiotic effects has been identified as a primary factor that kills invading bacilli. However, when target pathogens survive, ROS can induce DNA mutagenesis. ^30,83,84^ Thus, prolonged survival following a persister state, accompanied by regrowth through metabolic remodeling strategies, is directly associated with the development of populations harboring genetic mutation-mediated drug resistance.^12,85^ These metabolic adaptive strategies represent a mechanism underlying the accelerated emergence of MDR-TB.

A handful of investigations into the key metabolic remodeling strategies required for persister formation have begun. Our metabolomics studies, utilizing Mtb persisters collected from *in vitro* biofilm cultures or under hypoxic stress, have validated the functional importance of preexisting Mtb cell wall glycolipids as an alternative source for carbon nutrients.^13,22^ Bioinformatic analysis of the metabolomics data revealed that trehalose metabolism was one of the most significantly altered pathways compared to replicating Mtb. These findings suggest that Mtb persisters shift the catalytic direction of trehalose metabolism to biosynthesize intermediates in glycolysis and the PPP, a strategy termed the trehalose catalytic shift. Indeed, the ΔtreS of Mtb, which lacks this catalytic shift activity, exhibited hypersensitivity to first-line TB antibiotics such as INH and RIF. Trehalose serves as a structural component of Mtb cell wall glycolipids, such as TDM, which plays a crucial role in immunomodulatory interactions with the host immune system. Separately, sulfolipid-1 (SL-1) also contains trehalose as a core carbohydrate, and its function has recently been reported to be linked to the opportunistic transmission of Mtb bacilli to healthy individuals.^86^ Therefore, the trehalose catalytic shift plays multiple roles, including carbon storage, essential components for persister biology, antibiotic tolerance, immune evasion, and transmission.^38,87^

This study has revealed an additional role of the trehalose catalytic shift in accelerating the development of permanent MDR in Mtb. The bacilli maintain viability by forming persisters through this shift activity, even under bactericidal levels of oxidative stress. This process induces DNA mutagenesis via the activation of the trehalose catalytic shift. Notably, RIF single-resistant Mtb bacilli tend to exhibit increased levels of antibiotic tolerance against a second antibiotic, even without prior exposure. Consequently, this adaptive strategy can contribute to the emergence of MDR-TB. Furthermore, our study has shown that the trehalose catalytic shift enhances phenotypic heterogeneity. By inhibiting *treS* expression through CRISPR*i* or chemically deactivating TreS with ValA, a key enzyme in the trehalose catalytic shift, we observed a significant reduction in the emergence of DR mycobacterial mutants against clinically relevant TB antibiotics. Fascinatingly, our mathematical modeling has clarified that the trehalose catalytic shift uniquely facilitates persister formation and provides phenotypic stability, preventing persisters from reverting to the DS-state. This suggests that the frequency of DR mutant emergence is predominantly influenced by the extent of persister formation and its associated phenotypic heterogeneity and stability. We concluded this based upon our findings that the transition rates from persisters to permanent DR mutants were nearly identical between wildtype and ΔtreS. Therefore, the metabolic remodeling strategies that promote Mtb persister formation represent a novel source of antibiotic targets aimed not only at eliminating Mtb persisters but also at averting the onset of MDR-TB.

We have developed a novel technique to monitor mycobacterial phenotypic heterogeneity resulting from the active trehalose catalytic shift by labeling with an RMR-tre fluorogenic dye in conjunction with FACS analysis. In our recent report,^53^ we demonstrated that RMR-tre serves as a substrate of Ag85, an enzyme involved in the biosynthesis of TDM, at levels comparable to free trehalose. In this study, we identified that RMR-tre labeling intensity was proportional to the TreS activity (Fig. A-C). This is likely because mycobacterial cells with higher TreS direct a larger fraction of trehalose toward the biosynthesis of CCM intermediates, resulting in limited internal trehalose availability for TDM biosynthesis. Consequently, when exogenous RMR-tre is supplied, mycobacterial cells with greater TreS utilize more RMR-tre as a substrate for TDM, whereas TreS-deficient mycobacterial cells rely more on endogenous trehalose. In response to antibiotic treatment, Mtb exhibited an accumulation of RMR-tre^high^ bacilli, a subfraction displaying a labeling pattern similar to pTreS but absent in ItreS^SM^ or ΔtreS Mtb. This suggests that the activation of the trehalose catalytic shift leads to increased metabolic and phenotypic heterogeneity in Mtb, resulting in a newly formed population that exhibits tolerance to antibiotic effects. The Flux^RIF^ bacilli showed a higher abundance of the RMR-tre^high^ subfraction compared to naïve DS-bacilli, underscoring the essential role of the trehalose catalytic shift in the metabolic networks of DR-bacilli and thus underlying mechanisms that contribute to their cross-resistance to other antibiotics. Treatment with bactericidal concentrations of antibiotics caused a notable increase in the proportion of bacilli exhibiting high TreS activity. This effect may arise from the greater antibiotic susceptibility of the subfraction with low TreS activity or the alteration of their metabolic heterogeneity, which promotes the induction of TreS activity.

The exploration of metabolic strategies beyond the trehalose catalytic shift is warranted, as a significant portion of ItreS^SM^ or ΔtreS Mtb strains continue to develop drug-resistant mutations, albeit at a notably reduced rate. Consistent with the data obtained from Flux^RIF^ bacilli, metabolomics analysis of DR-TB clinical isolates revealed distinctly different metabolic activities involved in trehalose catalysis compared to DS-TB clinical isolates. These DR-TB clinical isolates exhibited biochemical similarities to Mtb persisters, characterized by dysregulated membrane bioenergetics and active glycolysis and the PPP which serve as alternate sources of energy and antioxidants. Additionally, they showed a reduced abundance of cell wall TDM, a proinflammatory ligand of Mtb, as part of an immune evasion strategy.^13,22,88^ The catabolic remodeling of TDM provides infected Mtb bacilli with a spatiotemporal advantage, allowing them to maintain their latent status without depending host nutrients or provoking excessive immune system stimulation. TB clinical isolates utilize host fatty acids or cholesterol as primary carbon sources, which require endergonic metabolic pathways such as the TCA cycle, glyoxylate shunt, methylmalonyl CoA pathway, and methylcitrate cycle, followed by gluconeogenic reactions. Gluconeogenesis primarily involves endergonic reactions that consume energy to biosynthesize carbohydrate intermediates. Therefore, the trehalose catalytic shift represents a catalytic advantage, enabling the exploitation of metabolic networks largely composed of exergonic reactions to support the energy needs and antioxidants requirements essential for Mtb persister biology, antibiotic tolerance, and the eventual survival of MDR mutants.

Labeling Flux^RIF^ bacilli with RMR-tre dye revealed that those carrying RRDR mutations exhibited significantly fewer Flux^RIF^ bacilli compared to the RRDR mutation-free Flux^RIF^ bacilli (Fig. S5B, C). Additionally, treatment with bactericidal antibiotics selectively eliminated the RMR-tre^low^ subfraction, a phenomenon that was more pronounced in the RRDR mutation-free Flux^RIF^ bacilli (Fig. S5D). This finding suggests that Flux^RIF^ bacilli lacking RRDR mutations may serve as a primary reservoir for the future development of DR mutations and the emergence of permanent MDR mutants. Consequently, these pre-resistant subfractions of bacilli may require a significantly higher level of trehalose catalytic shift to sustain their drug-resistant phenotype. Collectively, Mtb could achieve a permanent DR phenotype through the formation of Mtb persisters or pre-resistant bacilli by inducing the trehalose catalytic shift activity. Similar to carbapenem-resistant *Enterobacteriaceae* clinical isolates (CRE),^89^ Mtb persisters may revert to a DS-state once the effects of antibiotics diminish (Fig. 5A). In contrast, pre-resistant bacilli were found to be phenotypically stable, consistently managing their metabolic networks with a high level of trehalose catalytic shift. The functional relevance of the trehalose catalytic shift in the formation of Mtb persisters and/or pre-resistant bacilli was confirmed by genetically inactivating *treS* in Flux^RIF^ bacilli (Fig. 4D, E).

Consistent with previous findings, we observed that the greater frequency of MDR mutations in lineage 2 clinical strains such as HN878 is largely attributed to their increased trehalose catalytic shift activity. This enhanced activity is linked to the induced formation of persisters and pre-resistant bacilli. Our mathematical modeling suggests that the greater frequency of persister formation is phenotypically associated with increased likelihood of enhancing phenotypic heterogeneity and the emergence of MDR-TB. The cross-resistance study revealed that Mtb bacilli with elevated levels of trehalose catalytic shift are more susceptible to develop MDR-TB mutations. This research illuminates the metabolic basis for the increased frequency of MDR-TB cases in HN878 infections. Targeting the trehalose catalytic shift in HN878 offers a novel therapeutic strategy to enhance the efficacy of clinically relevant TB antibiotics by preventing both the formation of persisters and the emergence of MDR-TB cases. We recently demonstrated that certain trehalose structural analogues can disrupt Mtb persister formation and antibiotic tolerance by inhibiting TreS-centered trehalose catalytic shift activity, thereby enhancing the antimicrobial effects of INH or RIF.^39^ The potential antimicrobial synergy of these compounds with clinically relevant TB antibiotics against HN878 infection warrants further investigation.

## Materials & Methods

### Bacterial Strains, Culture Conditions, and Chemicals.

Drug-sensitive or drug-resistant *Mycobacterium smegmatis*, along with their CRISPR*i* strains including ItreS^SM^ and IotsA^SM^ were cultured at 37°C in Middlebrook 7H9 broth (m7H9) (Difco) or on Middlebrook 7H10 agar (m7H10) (Difco), supplemented with 0.04% Tyloxapol (for planktonic growth in m7H9 only), 0.5 g L^− 1^ BSA (Fraction V), 0.2% glycerol, 0.2% dextrose, and 0.085% NaCl. Kanamycin (50 μg/mL) was used to select for CRISPR*i* mutants. The *Mycobacterium tuberculosis* strains HN878, Erdman, H37Rv, and CDC1551, along with their CRISPR*i* strains (ItreS^HN^, ItreS^Erd^, ItreS^CDC^, and ItreS^Rv^) were cultured in a biosafety level 3 facility. Drug-sensitive (DS), rifampicin single-resistant (RSR), multidrug-resistant (MDR), extensively drug-resistant (XDR), and totally drug-resistant (TDR) TB clinical isolates were originally isolated from the sputum of TB patients at the National Masan Tuberculosis Hospital. We have complied all relevant ethical regulations for working with TB clinical isolates. When appropriate, cultures were supplemented with 10 mM sodium butyrate, 20 mM trehalose, 200 μM Validamycin A (ValA), 200 ng/mL Anhydrotetracycline (ATc), and 100X MIC rifampicin (RIF), isoniazid (INH), bedaquiline (BDQ), or d-cycloserine (DCS). These compounds were purchased from Sigma and Advanced ChemBlocks Inc. The experiments using the CRISPR*i* mutants were conducted with or without ATc. Without ATc treatment was included as a complement condition.

### Metabolite extraction and LC-MS analysis

*M. smegmatis*- or Mtb-laden filters used for metabolomics profiling were generated and incubated at 37°C for 5 days to reach the mid-log phase of growth.^21^ To prepare for filter-culture-based metabolomics profiling, *M. smegmatis* or Mtb grown on agar-supported filters were treated with Trehalose and/or ValA to expose larger inocula. *M. smegmatis* or Mtb-laden filters were metabolically quenched by immersing them in a precooled mixture of acetonitrile:methanol:H_2_O (40:40:20, v:v:v) at −40°C. Metabolites were extracted using mechanical lysis with 0.1-mm zirconia beads in a Precellys tissue homogenizer for 4 min at 6000 rpm, repeated twice under continuous cooling at or below 2°C. The lysates were clarified by centrifugation and then filtered through a 0.22-μm Spin-X column. The residual protein content of metabolite extracts was measured using a BCA protein assay kit (Thermo Scientific) to normalize the samples to cell biomass.

Extracted metabolites were separated using a Cogent Diamond Hydride type C column (gradient 3) with a mobile phase consisting of solvent A (ddH_2_O with 0.2% formic acid) and solvent B (acetonitrile with 0.2% formic acid). The mass spectrometer utilized was the Agilent Accurate Mass 6230 time of flight (TOF), coupled with an Agilent 1290 liquid chromatography (LC) system. Dynamic mass axis calibration was achieved through continuous infusion of a reference mass solution using an isocratic pump with a 100:1 splitter. This configuration resulted in mass errors of 5 ppm and mass resolution ranging from 10,000 to 25,000 over the m/z of 62–966 atomic mass units, with a dynamic range of 5 log_10_. Detected ions were identified as metabolites based on unique accurate mass-retention time identifiers for masses displaying the expected distribution of accompanying isotopomers. Metabolites were quantified using a calibration curve generated from chemical standards spiked into a homologous Mtb extract (1:10 diluted with metabolite extraction solution) to account for matrix-associated ion suppression effects. The abundance of metabolites was analyzed with Agilent Qualitative Analysis B.07.00 and Profinder B.07.00 software (Agilent Technologies), employing a mass tolerance of < 0.005 Da. Data analysis including clustered heatmap, hierarchical clustering, principal component analysis, volcano plot, and pathway enrichment analysis was conducted using MetaboAnalyst (ver. 6.0). All data obtained from metabolomics profiling represent the average of at least two independent triplicates.

### qRT-PCR analysis

*M. smegmatis* or Mtb strains were grown in m7H9 until they reached mid-log phase. Bacilli were harvested by adding an equal volume of guanidine thiocyanate buffer. Total RNA was extracted with TRIzol reagent and the Purelink RNA mini kit (Invitrogen), following the manufacturer’s instructions. Genomic DNA was removed from the samples using the Turbo DNA-free kit (Invitrogen). cDNA was synthesized from 500 ng of RNA using the iScript Kit (Bio-Rad) and quantitative PCR was performed with a C1000 Thermal Cycler. Primers and probes were designed using the PrimerQuest^™^ Tool (Integrated DNA Technologies), as detailed in Table S2. Fold changes were calculated based on ΔΔCt values normalized to the transcript levels of the *sigA* housekeeping gene and presented as log_2_ values.

### CRISPRi knockdown generation

Single guide RNA (sgRNA) was designed to target the 3’-end of the non-template strand open reading frame of the target genes, consisting of a ~ 20 nucleotide sequence located 5’ of an effective protospacer adjacent motif (PAM) sequence within the region. The PLJR962 *M. smegmatis* CRISPR*i* backbone plasmid was amplified in *E. coli*, selected with kanamycin (50 μg/mL), and then digested with *Bsm*BI restriction enzymes (NEB) before being cleaned and purified. The designed oligo primers (see Table S2) were annealed and ligated into the *Bsm*BI-digested plasmid backbone. Competent *M. smegmatis* cells were prepared by washing mid-log phase cultures multiple times in ice-cold 15% glycerol solution. The recombinant plasmid of interest was introduced into the competent cells via electroporation using a Pulse Controller II and Gene Pulser II (BioRad). Transformed liquid cultures were grown to mid-log phase and plated on m7H9 containing 50 μg/mL kanamycin to select for the recombinant colonies. These colonies were subsequently regrown in m7H9 containing kanamycin and incubated with ATc (at 200 ng/mL) for at least 1 day to induce target gene repression. The endogenous knockdown of the target genes of interest was confirmed by qRT-PCR.

### Luria–Delbrück Fluctuation Assay and analysis

The original classical fluctuation assay was modified for this study.^45^ For the generation of single-cell suspension of *M. smegmatis* wildtype and ItreS^SM^, cultures were diluted to an OD_595_ of 0.000005 in 200 μL within 96-well plates. The cultures were then regrown until they reach an OD_595_ of 0.7–1.0. A total of 100 μL from 60 randomly selected colonies were plated on m7H10 containing either 100 μg/mL RIF or 200 μg/mL INH. Spontaneous RIF- or INH-resistant colonies were counted after incubating for up to 10 days. To determine the total viable input cell number, the remaining 100 μL of the cultures were serially diluted and plated on m7H10 without antibiotics. Mutation rates were calculated using Lea-Coulson method (m/Nt where m, number of resistant colonies; Nt, total input).^90^

### Co-culture competition assay

The plasmids pTE-OX-GCT5 and pGMEH-p38-mRFP (Addgene) were transformed into wildtype *M. smegmatis* and ItreS^SM^ to generate wildtype::GFP and ItreS^SM^::RFP strains, respectively. Using these strains, we assessed the relative viability of wildtype and *treS* deficient strains after cyclic exposure to bactericidal concentrations of RIF or D-cycloserine (DCS). Briefly, equal volumes of mid-log phase cultures of wildtype::GFP and ItreS^SM^::RFP, both at an OD_595_ of ~ 0.7, were mixed to create G0 (untreated) culture. This culture was treated with 100 μg/mL RIF or DCS for 1 day, after which it was washed with PBS and resuspended in antibiotic-free m7H9. This resulted in a new culture at an OD_595_ of 0.05, which was then re-grown until it reached an OD_595_ of ~ 0.7, referred to as the G1 culture. The G1 culture was treated again with the same concentrations of RIF or DCS for 1 day, washed with PBS, resuspended in fresh m7H9, and re-grown until it reached an OD_595_ of ~ 0.7 to create the G2 culture. This procedure was repeated until we obtained the G5 culture. Flow cytometry was employed to detect the relative abundance of wildtype and ItreS^SM^ during the generation of G0 to G5 cultures.

### Spot assay

Flux^RIF^ and Flux^INH^ cultures at mid-log phase were diluted to an OD_595_ of 0.1. Five 10-fold serial dilutions were then prepared, and 2 μL of each culture were spotted onto m7H10 containing 8–100 μg/mL RIF, 4–32 μg/mL INH, or 0.0075–0.03 μg/mL BDQ. The plates were incubated at 37°C until colonies formed.

### RRDR sequencing of lab made RIF resistant M. smegmatis

The *rpoB* gene sequences of the Flux^RIF^ mutants were compared to the reference *rpoB* genes for wildtype *M. smegmatis*.^91^ ApE plasmid-editing software was utilized to identify mutations. Flux^RIF^ strains were streaked onto antibiotic-free LB agar plates and incubated at 37°C overnight to isolate colonies for sequencing. The RRDR (RIF Resistance Determining Region) of the *rpoB* gene was assessed using Sanger Sequencing (Quintara Biosciences) with the following primers: Msmeg-*rpoB*-fwd (5’-gctgatccagaaccagatcc-3’) and Msmeg-*rpoB*-rev (5’-gatgacaccggtcttgtcg-3’).

### Membrane bioenergetics – membrane potential, NAD/NADH, ATP

For membrane potential (ΔΨm) measurement, cultures were grown in m7H9 to mid-log phase and concentrated to an OD_595_ of ~ 1.0 in fresh m7H9. The cultures were treated with 15 μM DiOC_2_ and incubated for 40 min at 37°C, followed by washing with PBS to remove any extracellular dye. As a positive control for membrane depolarization, one culture was treated with 5 μM of the protonophore carbonyl-cyanide 3-chlorophenylhydrazone (cccp) (Invitrogen), while PBS served as a vehicle control. The assay was conducted in black clear-bottom 96-well plates (Costar), and a SpectraMax M4 spectrofluorimeter (Molecular Devices) was used to measure green fluorescence (488 nm/530 nm) and shifts to red fluorescence (488 nm/610 nm). ΔΨm was calculated as the ratio of red fluorescence to green fluorescence, with each condition measured in triplicate.

To assess intrabacterial ATP and NADH/NAD levels, *M. smegmatis*-laden filters generated for the metabolomics profiling were used. Intrabacterial ATP concentrations were measured using the BacTiter Glo Microbial Cell Viability Assay kit, following the manufacturer’s instructions (Promega). NAD and NADH concentrations were measured using the FluroNAD/NADH detection kit, also following the manufacturer’s instructions (Cell Technology). Bacterial metabolism was rapidly quenched by plunging the filters into the first solvent in each kit.

### RMR-tre labeling and Flow cytometry

RMR-tre was synthesized as previously reported and characterized by nuclear magnetic resonance (NMR) spectroscopy.^53^ Wildtype *M. smegmatis*, ItreS^SM^, pTreS^SM^, Flux^RIF^, or ItreS^Flux^ cultures in mid-log phase were treated with 1X MIC RIF for 1 day. The cultures were then stained with RMR-tre fluorogenic dye at a final concentration of 10 μM and incubated at 37°C for 1 hour. Following incubation, the cultures were sorted using a flow cytometer (Attune NxT, Thermo Fisher Scientific) and the reported values represent the gated cell fraction. Data were exported from the flow cytometry cytometer and analyzed using FlowJo software (BD Biosciences). Error bars represents the standard deviation from biological replicates.

### Mathematical modeling

The classical fluctuation assay was modified (Fig. S6A). To generate single lineage bacilli of *M. smegmatis* wildtype and ItreS^SM^, cultures were diluted to an OD_595_ of 0.00005 in 200 μL and dispensed into 96-well plates. The cultures were regrown until they reach an OD_595_ of 0.7–1.0. A total of 100 μL from 60 randomly selected wells were plated on m7H10 containing 100 μg/mL RIF, and spontaneous RIF-resistant colonies were counted after incubating for 10 days. To determine the total viable input cell number, the remaining 100 μL of cultures were serially diluted and plated on antibiotic-free m7H10. To infer the reversible switching rates between drug-sensitive and drug-tolerant states, we utilized recent mathematical work on calculating these rates from the fluctuation assay. The variability $CV_{N_{T}}^{2}$ – quantified by the coefficient of variation (standard deviation divided by the mean) – is given by:

(1) $$CV_{N_{T}}^{2}\times f=\frac{2T_{\mathrm{on}}e^{T-\frac{2T}{T_{on}}}-2-T_{\mathrm{on}}}{\left(2e^{T}-1\right)\left(T_{\mathrm{on}}-2\right)}$$

where T is the time (normalized to the bacterial doubling time) for which single bacillus colonies are expanded, $T_{on}$ is the average time (also normalized to the bacterial doubling time) spent in the drug-tolerant state, and f is the fraction of bacilli that are persisters.^59,60^

Upon long-term antibiotic exposure, each drug-tolerant bacilli irreversibly transition to a permanent drug-resistant state with a small probability p≪1. Conditioned on the number of drug-tolerant bacilli $N_{T}$, the number of drug-resistant colonies $N_{R}$ will follow a binomial distribution. The clone-to-clone variation in $N_{R}$ is given by:

(2) $$CV_{N_{R}}^{2}=CV_{N_{T}}^{2}+\frac{1-p}{{\overset{‾}{N}}_{R}}\approx CV_{N_{T}}^{2}+\frac{1}{{\overset{‾}{N}}_{R}}$$

where ${\overset{‾}{N}}_{R}$ and $CV_{N_{R}}$ denote the average and the coefficient of variation of $N_{R}$, respectively. Using (2), measurements of the number of colonies growing on antibiotics across single bacillus lineages on can estimate $CV_{N_{T}}^{2}$:

$$CV_{N_{T}}^{2}\approx CV_{N_{R}}^{2}-\frac{1}{{\overset{‾}{N}}_{R}}$$

This estimate can then be used to estimate $T_{on}$ from (1). Analyzing the fluctuation assay data on $N_{R}$ from 60 single-cell lineages, we estimate:

$$CV_{N_{T}}^{2}\approx 2.41\pm 1.5$$

for the wildtype genotype, where ± denoted the 95% confidence interval as estimated by bootstrapping. Using this estimate in (1) with T=30 (i.e., each single bacillus was expanded for 30 cell generations before plating on RIF-containing plates) and the average frequency of drug-tolerant persisters as $f=10^{-3}$, we obtain the transient heritability of wildtype persister state to be $T_{\mathrm{on}}=9.6\pm 0.75$ generations. Our data show that ItreS^SM^ has approximately six-fold fewer colonies growing on antibiotics compared to wildtype. Assuming the same value of p for both genotypes, this decrease could result from a six-fold lower persister-formation rate in ItreS^SM^ while maintaining $T_{on}$ at the same level. Analyzing the fluctuation assay data, we obtain:

$$CV_{N_{T}}^{2}\approx 2.52\pm 2.4$$

for the ItreS^SM^. Using this value with $f=\frac{10^{-3}}{6}$ we estimate:

$$T_{on}=7.4\pm 1.1$$

generations from (1), which represents approximately a 20% decrease compared to its value in wildtype. This slight decrease alone cannot account for the six-fold change in $N_{R}$, which must result from a six-fold decrease in the transition rate from the drug-sensitive to the drug-tolerant state. Overall, the fluctuation assay data suggest that ItreS^SM^ persisters are slightly more unstable and revert to being drug-sensitive more quickly. The observed difference in $N_{R}$ is primarily due to a six-fold lower rate of persister formation in ItreS^SM^ compared to wildtype.

### Antibiotic permeability experiment/ EtBr permeability assay

*M. smegmatis* wildtype, Flux^RIF^, or Flux^INH^ in mid-log phase were incubated with 1X MIC RIF at 37°C. Bacteria were harvested at 0, 2, 4, 24, and 48 hrs and CFUs were determined by plating serial dilutions on m7H10. The cell-free supernatant was collected by filtering through a 0.22 μm filter. RIF was extracted by adding a precooled solution of LC-MS grade acetonitrile:methanl:H_2_O_2_ (40:40:20) −40°C. RIF detection and quantification were performed using a Cogent Diamond Hydride Type C column (Microsolve Technologies) coupled with an Agilent Accurate Mass 6230 TOF and an Agilent 1290 Liquid Chromatography system, as previously reported.^21,23,92^ The intrabacterial RIF concentration was calculated as $[RIF{]}_{\text{drug}\;\text{only}}-[RIF{]}_{\text{filtrate}}$. Three biological replicates were tested per group.

*M. smegmatis* wildtype and drug-resistant strains were grown in m7H9 until an OD_595_ of 0.7 was reached. Final 5 mL cultures were centrifuged at 13,000 rpm for 3 min, after which the supernatant was discarded and the pellet was washed with PBS. The OD_595_ was then adjusted to 0.4 with PBS, and glucose was added to a final concentration of 0.4%. Ethidium bromide (EtBr) was added at a concentration of 8 mg/mL, and 100 μL aliquots were transferred to each well of black, clear-bottom 96-well plates (Costar). A SpectraMax M5 spectrofluorometer (Molecular Devices) was used to measure fluorescence, with excitation and emission wavelength set to 530 nm and _595_ nm, respectively. Fluorescence data were acquired every 60 sec for 60 min.

### MPN (the most probable number) method to detect the total persister bacilli

The MPN method was conducted as previously reported.^72^ Briefly, HN878, H37Rv, Erdman, and CDC1551 strains in mid-log phase were grown in m7H9, harvested by centrifugation at 5,000 rpm for 8 min, and washed twice with PBS. The cultures were then centrifuged at 800 rpm for 8 min to generate a single-cell suspension. The supernatant was transferred to a separate tube and diluted with PBS to achieve an OD_595_ of 0.1. A CFU assay was used for the inoculum quantification. A total of 20 mL of the single-cell suspension was prepared for each condition, transferred to a vented flask, and incubated at 37°C for 2 weeks. The starved cultures were then split into two 8 mL cultures in vented flasks and treated with either 100 μg/mL RIF with or without 200 μM Validamycin A (ValA). The flasks were incubated at 37°C for 5 days. After incubation, cultures were harvested by centrifugation at 5,000 rpm for 8 min, washed with PBS, and resuspended in 1 mL PBS. A MPN method was then performed by diluting 15 μL of the 1 mL culture in 135 μL of m7H9 in 96-well plates. Ten-fold serial dilutions were carried out in the 96-well plates, which were stored at 37°C. The OD_595_ was measured after 3 weeks and again 5 weeks. The remaining culture from the assay was used for a CFU assay, with dilutions between ranging from 10^− 5^ and 10^− 1^. ItreS^HN^ and ItreS^CDC^ treated with or without ATc were also included to conduct the MPN assay.

### Statistical analysis

All data were analyzed by Prism (v10.0; GraphPad Software). Significant differences were calculated by unpaired Student’s t-test and one- or two-way ANOVA using multiple-comparison tests as specified or the nonparametric Mann-Whitney test for skewed data. P values less than 0.05 were considered statistically significant.

## Data Availability

All data generated or analyzed during this study are included in this published article and its supplementary files. The raw metabolomics datasets have been deposited in MetaboLights.
